# Defining biomarkers in oral cancer according to smoking and drinking status

**DOI:** 10.3389/fonc.2022.1068979

**Published:** 2023-01-11

**Authors:** Juliette Rochefort, Ioannis Karagiannidis, Claude Baillou, Lisa Belin, Maude Guillot-Delost, Rodney Macedo, Aline Le Moignic, Véronique Mateo, Patrick Soussan, Isabelle Brocheriou, Jean-Luc Teillaud, Marie-Caroline Dieu-Nosjean, Chloé Bertolus, Francois Michel Lemoine, Géraldine Lescaille

**Affiliations:** ^1^ Sorbonne Université, Inserm U.1135, Center of Immunology and Infectious Diseases (Centre d'Immunologie et des Maladies Infectieuses, CIMI-Paris), Paris, France; ^2^ Assistance Publique-Hôpitaux de Paris (AP-HP), Groupe hospitalier Pitié-Salpêtrière, Department of Odontology, Paris, France; ^3^ Faculty of Odontology Université Paris Cité, Paris, France; ^4^ Sorbonne Université, Inserm, Institut Pierre Louis d'Épidémiologie et de Santé Publique, AP-HP, Hôpitaux Universitaires Pitié-Salpêtrière - Charles Foix, Département de Santé Publique, Paris, France; ^5^ AP-HP, Hôpital Tenon, Department of Virology, Paris, France; ^6^ AP-HP, Groupe hospitalier Pitié-Salpêtrière, Department of Pathology, Paris, France; ^7^ AP-HP, Groupe hospitalier Pitié-Salpêtrière, Department of Maxillo-Facial Surgery, Paris, France; ^8^ AP-HP, Groupe hospitalier Pitié-Salpêtrière, Department of Immunology, Paris, France

**Keywords:** oral cancer, prognostic biomarker, tumor microenvironment, oral squamous cell carcinoma, non-smoker, non-drinker

## Abstract

**Introduction:**

Oral Squamous Cell Carcinomas (OSCC) are mostly related to tobacco consumption eventually associated to alcohol (Smoker/Drinker patients: SD), but 25-30% of the patients have no identified risk factors (Non-Smoker/Non-Drinker patients: NSND). We hypothesized that these patients have distinguishable immune profiles that could be useful for prognosis.

**Materials and Methods:**

Cells present in immune tumor microenvironment (TME) and blood from 87 OSCC HPV-negative patients were analyzed using a multiparameter flow cytometry assay, in a prospective case-control study. Cytokine levels in tumor supernatants and blood were determined by a cytometric bead array (CBA) assay.

**Results:**

Normal gingiva and blood from healthy donors (HD) were used as controls. A significant increase of granulocytes (p<0.05 for blood), of monocytes-macrophages (p<0.01 for blood) and of CD4^+^ T cells expressing CD45RO and CCR6 (p<0.001 for blood; p<0.0001 for TME) as well as higher levels of IL-6 (p<0.01 for sera, p<0.05 for tumor supernatant) were observed in SD patients as compared to NSND OSCC patients and HD. High percentages of CD4^+^ T cells expressing CD45RO and CCR6 cells in tumor tissue (p=0.05) and blood (p=0.05) of SD OSCC patients were also associated with a poorer prognosis while a high percentage of regulatory T cells (Treg) in tumor tissue was associated with a more favorable prognostic factor (p=0.05). Also, a higher percentage of blood CD8^+^ T lymphocytes among CD45^+^ cells in NSND patients was associated with a better disease-free survival (p=0.004).

**Conclusion:**

Granulocytes, monocytes-macrophages, and CD4^+^ T cells expressing CD45RO and CCR6 in blood and TME as well as serum IL-6 can therefore distinguish OSCC SD and NSND patients. Quantifying the proportion of CD4^+^ T cells expressing CD45RO and CCR6 and of Treg in SD patients and CD8^+^ T cells in NSND patients could help defining the prognostic of OSCC patients.

## 1 Introduction

Oral squamous cell carcinomas (OSCC) have a poor prognostic. The main risk factors are tobacco consumption associated or not to alcohol intake (Smoker/Drinker patients: SD) (1). However, OSCC are also diagnosed in non-smoker and non-drinker patients (NSND) (2), in particular in young patients (<40-years old) (3, 4), notably for the tongue in females (5, 6).

OSCC in NSND patients are epidemiologically different from SD patients (4, 6). For the young NSND patients, a direct oncogenic role of HPV has not been demonstrated (7–9), unlike oropharyngeal SCC (10), although the gene expression profiles are potentially compatible with a “hit and run” viral origin (11, 12). NSND and SD patients with OSCC can be also discriminated by differences in their immune tumor microenvironment (TME) (13). However, only few studies have analyzed immune cells present within the TME and/or the blood of Head and Neck Squamous Cell Carcinoma (HNSCC) patients and whether the NSND and SD OSCC patients exhibit different immune characteristics (14–16). In HNSCC smoker patients, a marked immunosuppression is revealed by a higher mutational smoking signature associated with lower immune infiltrates and a differential gene expression profile (14). Smokers patients with human papillomavirus (HPV)-negative OSCC exhibit a decrease of CD8^+^ T cells and PD-L1^+^ cells (15). Other studies have reported a decrease of dendritic cells (DCs) in the TME of smokers with OSCC as compared to non-smokers (16).

Moreover, tumor infiltrating lymphocytes (TILs) such as CD8^+^ T lymphocytes and regulatory T cells (Treg) have been associated with a favorable outcome in oropharyngeal cancers (17, 18). Their impact on the prognosis in OSCC patients is still unclear, even when an elevated neutrophil-to-lymphocyte ratio (NLR) and a platelet-to-lymphocyte ratio (PLR) are associated with a negative survival (19). Interestingly, a higher proportion of T helper 17 cells (Th17 cells) among blood cells has been reported in OSCC patients (20). However, their role as pro- or anti-tumor effector cells is a matter of debate (21). Of note, it is still unclear whether the immune cell subsets infiltrating the tumor or circulating in blood of OSCC patients represent biomarkers related to either SD or NSND status and can be differentially associated with prognosis in these two groups of patients. Thus, in the present study, we investigated whether OSCC in SD and NSND patients exhibit distinguishable immune profiles that could make it possible to define *biomarkers* of interest. We have therefore analyzed immune cell subsets in the blood and TME of OSCC patients separated depending on their risk factors.

## 2 Materials and methods

### 2.1 Patients and healthy donors

87 primary OSCC patients (63 males and 24 females) were recruited before any treatment in the department of Maxillo-facial Surgery (Pitié-Salpêtrière hospital, Paris, France). Clinicopathological characteristics were collected and are presented in **Table 1**. Patients were separated into non-smokers/non-drinkers (28 NSND) and smokers/drinkers (59 SD). As opposed to SD patients, NSND were defined as strictly non-smokers (no consumption of any pack per year) or having stopped smoking at least 15 years prior to the date of diagnosis, regardless of the amount smoked and as (ii) non-drinkers or having light/occasional alcohol consumption or having stopped drinking at least 15 years prior to the date of diagnosis, regardless of initial alcohol consumption. Among the 12 NSND patients belonging to the >70-year-old decade and beyond (**Table 1**), two of them stopped smoking 20 and 35 years prior to the date of diagnosis, all the others being strictly non-smokers. 49/87 patients had no medical history and were not under medical treatment related to other systemic diseases (13 NSND and 36 SD). For other patients, the three most common diseases were diabetes (5 NSND and 3 SD) hypertension (9 NSND and 7 SD), and Chronic Obstructive Pulmonary Disease (4 SD). Tumor staging was assessed according to the Union for International Cancer Control’s (UICC) tumor-node-metastasis criteria (22). 71 NSND and SD healthy donors (HD) were recruited from the Odontology department and from the department of Addictology (Pitié-Salpêtrière hospital). Age and gender of HD are depicted in **Supplementary Table S1**. Normal gingival tissue samples were obtained from 18 HD (9 NSND, 9 SD) and peripheral blood from 53 HD (19 NSND, 34 SD) was collected. All gingival samples were collected from sites without clinical signs of gingival inflammation. Surgery indications were orthodontic indication or root fracture. The piece of gingiva was removed from the attached gingiva and not from the free gingiva to avoid any source of local inflammation. All participants were recruited between 2013 and 2017.

**Table 1:** Clinical features of OSCC patients and differences between NSND and SD OSCC patients.

Characteristics		OSCC^a^ patients n = 87	NSND^b^ patients n = 28	SD^c^ patients n = 59	p-value
Gender	Male	63 (72.41%)	16 (57.14%)	47 (79.66%)	0.028
	Female	24 (27.59%)	12 (42.86%)	12 (20.34%)	
Age	Min/Max^d^	28/87	30/87	28/81	0.058
	Med^e^ [IQR^f^]	62 [52.5-69]	67 [51.25-77.25]	60 [52.5-66.5]	
	Mean (std)	59.91 ± 13.72	63.11 ± 18.03	58.39 ± 10.97	
	<50 years	19 (21.84%)	7 (25%)	12 (20.34%)	0.0016
	50-70 years	49 (56.32%)	9 (32.14%)	40 (67.8%)	
	>70 years	19 (21.84%)	12 (42.86%)	7 (11.86%)	
Smoking	No	28 (32.18%)	28 (100%)	0 (0%)	<0.0001
	Yes	59 (67.82%)	0 (0%)	59 (100%)	
Drinking	No	42 (48.27%)	28 (100%)	14 (23.72%)^g^	<0.0001
	Yes	45 (51.72%)	0 (0%)	45 (76.27%)	
Localizations	Gingiva	30 (34.48%)	10 (35.71%)	20 (33.9%)	0.185
	Tongue	28 (32.18%)	9 (32.14%)	19 (32.2%)	
	FTM^h^	21 (24.14%)	4 (14.29%)	17 (28.81%)	
	Others^i^	8 (9.2%)	5 (17.86%)	3 (5.08%)	
Nodal	No	39 (44.82%)	14 (50%)	25 (42.37%)	0.814
	Yes	48 (54.17%)	14 (50%)	34 (57.62%)	
UICC^j^ Stage	I	18 (20.69%)	7 (25%)	11 (18.64%)	0.575
	II	9 (10.34%)	3 (10.71%)	6 (10.17%)	
	III	6 (6.9%)	3 (10.71%)	3 (5.09%)	
	IV	54 (62.07%)	15 (53.57%)	39 (66.1%)	

^a^OSCC, oral squamous cell carcinoma; ^b^NSND, Non-Smoker/Non-Drinker; ^c^SD, Smoker/Drinker; ^d^Min, minimum/Max: maximum; ^e^Med, median; ^f^IQR, Inter quartile Range; ^g^Not all smokers are drinkers; ^h^FTM, Floor of the mouth; ^i^Other localizations are cheek, palate, lips; ^j^UICC, Union for International Cancer Control’s. Data are presented as numbers and (relative percentage). Qualitative variable analyses were performed by a chi-square test and quantitative variable analyses by a Student t-test, comparing NSND and SD patients. Non-parametric tests (Wilcoxon or Fisher tests) were used when appropriate. p-value ≤0.05 was considered as significant.

### 2.2 Samples from patients and healthy donors

Peripheral blood was collected from the 87 OSCC patients before surgery. Fresh tumor samples (collected from tumor edge) of 44 of the 87 OSCC patients (14 NSND; 30 SD) were obtained after surgical resection or biopsy. Gingival tissues (non-tumor tissue controls) were obtained from 18 HD (9 NSND; 9 SD) undergoing preventive tooth extraction. Written informed consents were obtained and the study was approved by the institutional review board and by the Ethics Committee of Inserm (n°19-564) and conducted in accordance with the Declaration of Helsinki.

### 2.3 HPV analysis in tumor samples

Tumor fragments were obtained by punching in the tumoral zones of paraffin-embedded tumor blocks (Department of Pathology, Pitié-Salpêtrière hospital). HPV status was determined using the INNO-LiPA HPV Genotyping Extra II (Fujirebio, Les Ulis, France) according to the manufacturer’s instructions.

### 2.4 Cell isolation and multiparameter flow cytometry analysis

Single cell suspensions were obtained after non-enzymatic digestion of fresh tumor tissues or from gingival tissue using the Cell Recovery Solution (Corning, Boulogne-Billancourt, France) at 4°C for 1 h. Cells were stained with Live/Dead-APCH7™ (Invitrogen - Thermo Fisher Scientific, Illkirch, France) and stained with labeled monoclonal antibodies (mAbs) (**Supplementary Table S2**) or isotype-matched mAbs used as controls according to manufacturer’s instructions. After cell surface staining, red blood cells were lysed.

For Foxp3 intracellular staining, cells were fixed and permeabilized with Foxp3/Transcription Factors Staining Buffer Set (eBioscience – Thermo Fisher Scientific) and then stained with a Foxp3 mAb. Acquisition and data analyses were performed using a LSRII flow cytometer (Becton Dickinson, Le Pont de Claix, France), FlowJo software (TreeStar, Ashland, OR, USA) and Kaluza software (Beckman Coulter, Roissy CDG, France). The immune cell subsets analyzed were analyzed using the following cell surface markers: Leukocytes: CD45^+^ cells; Granulocytes (Gr) (eosinophils-neutrophils): CD45^+^CD15^+^CD11b^+^ cells; Monocytes-macrophages (Mo-Mϕ): CD45^+^CD14^+^ cells; B lymphocytes: CD45^+^CD19^+^ cells; CD4^+^ T cells: CD45^+^CD3^+^CD4^+^ cells; CD8^+^ T cells: CD45^+^CD3^+^CD8^+^ cells; Treg: CD45^+^CD4^+^ CD25^high^CD127^low^Foxp3^+^ cells; CD4^+^ T cell subset (able to polarize towards a Th17 phenotype) (23, 24): CD45RO^+^CCR6^+^CD4^+^CD45^+^ cells. The gating strategy is shown in **Supplementary Figure S1**.

### 2.5 Detection of IL-17 in peripheral blood T lymphocytes

2x10^6^ Peripheral blood mononuclear cells (PBMC) isolated from 22 NSND and 15 SD OSCC patients were stimulated in RPMI 1640 medium containing 10% heat-inactivated fœtal calf serum with phorbol 12-myristate 13-acetate (PMA) (50 ng/mL) and ionomycin (1 µM) in the presence of Brefeldin A (10 µg/mL) (Sigma-Aldrich, Saint-Quentin-Fallavier, France) for 4 h. Cells were stained with anti-CD3 and anti-CD4 labelled mAbs, fixed and permeabilized with the fixation/permeabilization buffer (BD Biosciences). An intracellular antibody staining of IL-17 (**Supplementary Table S2**) was then performed according to manufacturer’s instructions. Acquisition and data analyses were performed as described above.

### 2.6 Measurement of cytokines in tumor supernatants and sera from OSCC patients

Supernatants were obtained by incubating 25-30 mg of 11 (4 NSND, 7 SD) tumor fragments in 250 µL of RPMI medium supplemented with glutamine and penicillin/streptomycin for 24 h. Sera samples were obtained from 26 OSCC patients (11 NSND and 15 SD) and 18 HD (5 NSND, 13 SD). Samples were frozen at -80°C. Cytokine levels were measured in tumor supernatants and sera by a cytometric bead array (CBA) assay using the human Th1/Th2/Th17 Cytokine Kit (BD Biosciences, Le Pont de Claix, France). Data were collected using a LSRII flow cytometer (Becton Dickinson) and then processed for analysis with the FCAP Array software (version 3.0, BD Biosciences).

### 2.7 Statistical analysis/data analysis

Qualitative variable analyses were performed by a chi-square test and quantitative variable analyses (means and/or medians with variances and/or range) by a Student t-test, comparing NSND and SD patients. In some experiments, the absolute number of cells was calculated because a change in the cell percentage could be due to the expansion or the decrease of other cells rather than a change in the absolute number of the cells being investigated. Non-parametric tests (Wilcoxon or Fisher tests) were used when appropriate. To evaluate if differences were significantly associated with risk factors independently of clinical parameters, an ANOVA regression model was used. Disease-free survival (DFS) was defined from the date of the initial diagnosis to the date of progression, recurrence, death, or at their last known contact date. Kaplan-Meier method and Cox regression model were used to analyze univariable and multivariable outcomes based on high or low levels according to the median of immune biomarkers. The Log-Rank test was used to calculate the differences observed between the groups of patients. Values are presented as mean ± SEM. Statistical analysis was performed with R Version 3.4.1. software.

## 3 Results

### 3.1 Clinical characteristics of NSND and SD OSCC patients are different

**Figure 1:**
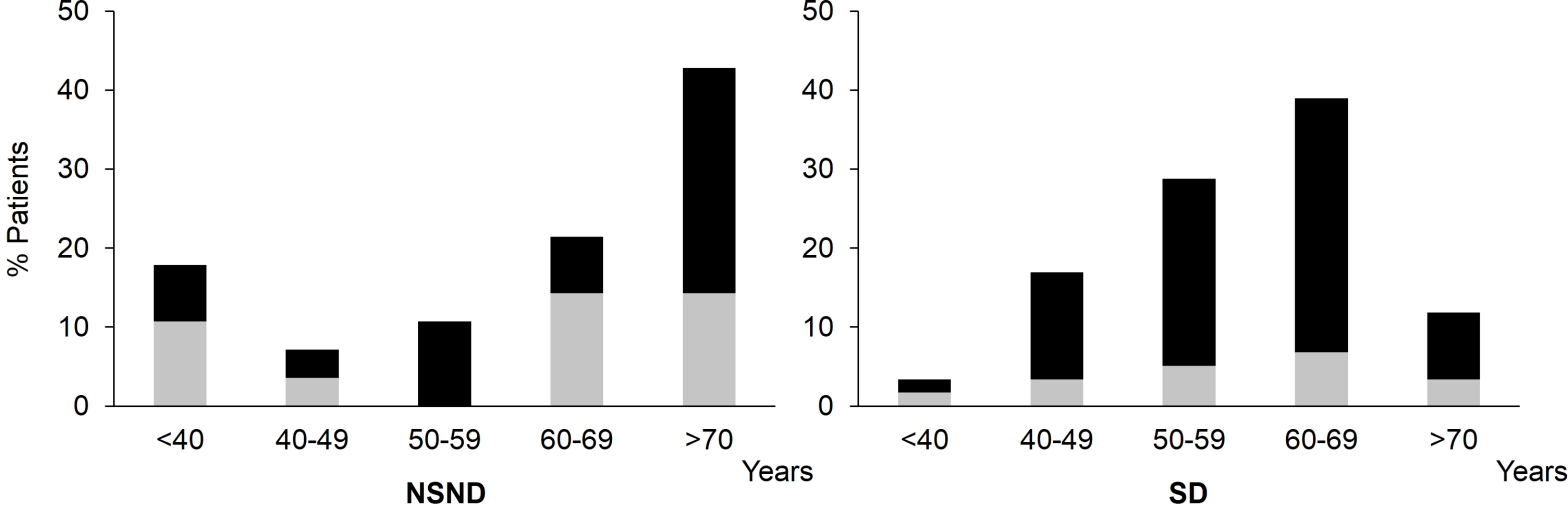
Age and gender distribution of NSND and SD OSCC patients depending on their risk factors. □: female, ■: male; OSCC, Oral Squamous Cell Carcinoma; NSND, Non-Smoker/Non-Drinker; SD, Smoker/Drinker.

The cohort of 87 HPV-negative OSCC patients was separated into 59 SD and 28 NSND (**Table 1**). The gender and age distribution of the two groups were different (**Figure 1** and **Table 1**). Although we did not observe statistical differences between SD and NSND patients for age ranking, patients between 50-70 years old were twice more frequent in the SD group than in the NSND group. Patients over 70 years were 3.6-fold more frequent in the NSND group that in the SD group. Below 50 years old, NSND and SD patients were rather equally distributed (**Figure 1** and **Table 1**).

Tumors from gingiva and tongue represented about 2/3 of all OSCC in both groups of patients (**Table 1**). However, tumors located in the floor of the mouth were twice more frequent in SD patients than in NSND patients. No statistical difference in the staging of the lesions or between non-nodal/nodal cancers was observed between NSND and SD patients. In term of disease-free survival (DFS), no statistical difference was found between the two groups of patients (data not shown).

### 3.2 Tumor microenvironment and blood from NSND and SD patients exhibit different immune composition

**Figure 2:**
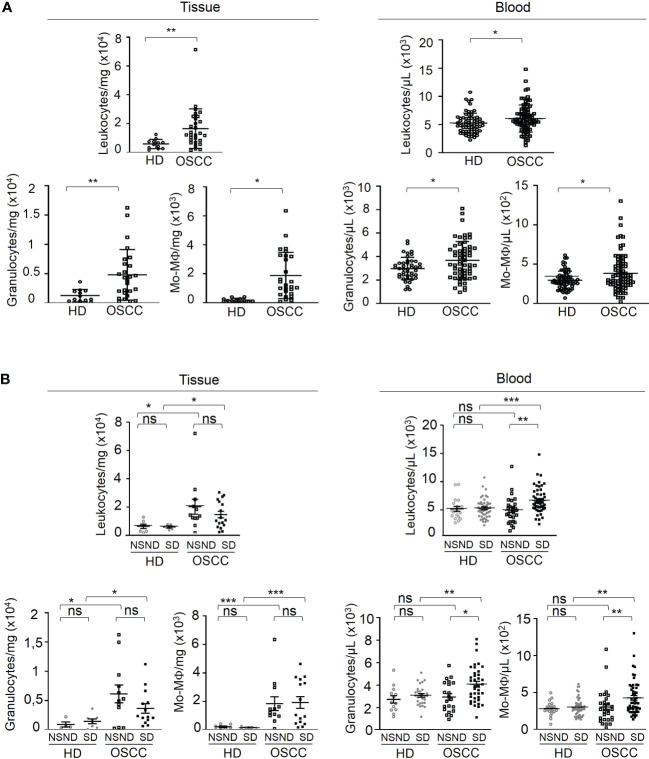
Analysis of the innate immune contents of tumor tissue and blood samples from OSCC patients and healthy donors according to risk factors (SD *vs* NSND). Data are presented either as absolute number of cells per mg of tumor or gingival tissue, or as absolute number of cells per µL of blood. **(A)** Analysis of leukocytes, granulocytes and monocytes-macrophages (Mo-Mϕ) of OSCC patients and healthy donors (HD); data from OSCC patients (□, 44 tumors and 87 blood samples from OSCC patients) were compared to those obtained from healthy donors (○, 18 normal gingival tissues and 53 blood samples). **(B)** Analysis of leukocytes, granulocytes and monocytes-macrophages (Mo-Mϕ) of OSCC patients and healthy donors depending on risk factors exposition. Data from SD (■) and NSND (□) OSCC patients were compared to those obtained from SD (●) and NSND (○) healthy donors. 14 tumors and 28 blood samples from NSND OSSC patients and from 30 tumors and 59 blood samples from SD OSCC patients, and 9 normal gingival tissues and 19 blood samples from NSND healthy donors and from 9 normal gingival tissues and 34 blood samples from SD healthy donors were analyzed by flow cytometry. For statistical analysis, a Mann-Whitney test was used. Bars represent the standard error of the mean (SEM). *p ≤ 0.05; **p ≤ 0.01; *** p ≤ 0.001; ns: not significant.

Immune cells of the TME and blood from patients were analyzed by multiparameter flow cytometry (**Figure 2**). For all OSCC patients, a significant increase of leukocytes was observed as compared to tissues and blood from HD (**Figure 2A**, top left and right panels) with a parallel increase of granulocytes and monocytes-macrophages observed in TME (**Figure 2A**, lower left panels) and in the blood (**Figure 2A**, lower right panels) of OSCC patients as compared to HD.

Moreover, leukocytes, granulocytes and monocytes-macrophages (Mo-Mϕ) blood counts from SD OSCC patients were significantly increased as compared to NSND (**Figure 2B**, right upper and lower panels). In TME, the counts of these cells were not statistically different between the two groups of patients but remained strongly increased compared to tissues from HD with similar risk factors (**Figure 2B**, upper and lower left panels).

**Figure 3:**
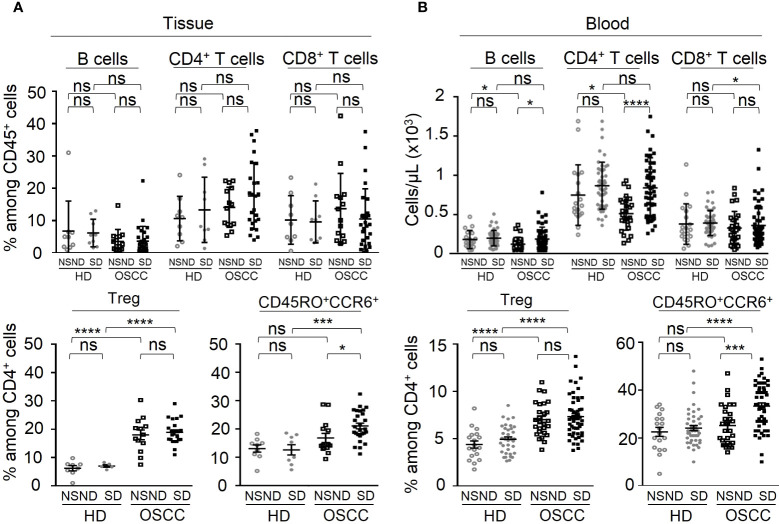
Analysis of the adaptive immune contents of tumor tissue and blood samples from OSCC patients and healthy donors according to risk factors (SD *vs* NSND). Data are presented either as percentages of cells among CD45^+^ cells or CD4^+^ T cells, or as absolute number of cells per µL of blood. **(A)** Tissue analysis and **(B)** Blood analysis, of B lymphocytes, CD4^+^ and CD8^+^ T cells among CD45^+^ cells (upper panels), and of Treg and CD45RO^+^CCR6^+^ cells among CD4^+^ T cells (lower panels) of OSCC patients and healthy donors depending on risk factors exposition. Data from SD (■) and NSND (□) patients were compared to those obtained from SD (●) and NSND (○) healthy donors (14 tumors and 28 blood samples from NSND OSSC patients; 30 tumors and 59 blood samples from SD OSCC patients; 9 normal gingival tissues and 19 blood samples from NSND healthy donors; 9 normal gingival tissues and 34 blood samples from SD healthy donors). For statistical analysis, a Mann-Whitney test was used. Bars represent the standard error of the mean (SEM). *p ≤ 0.05; *** p ≤ 0.001; ****p ≤ 0.0001; ns: not significant.

The percentages of B cells, CD4^+^ and CD8^+^ T cells in tissue were not different between patients and healthy donors (**Figure 3**, upper left panel). A significant decrease of the absolute numbers of blood CD4^+^ T cells and B cells was observed in NSND patients as compared to SD patients and to NSND healthy donors (**Figure 3**, upper right panel). The absolute number of circulating CD8^+^ T cells from SD patients was slightly, albeit significantly (p = 0.03), decreased as compared to SD healthy donor group (**Figure 3**, upper right panel).

**Figure 4:**
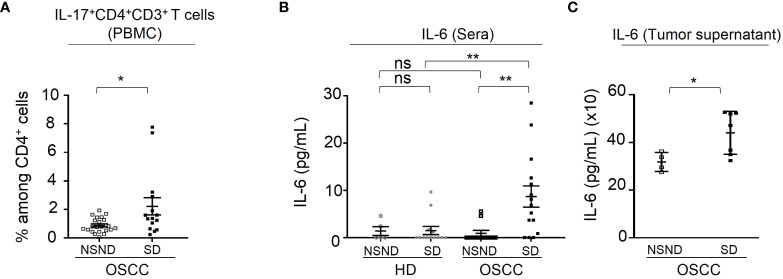
Analysis of IL-17^+^CD4^+^CD3^+^ T cells and of IL-6 production. **(A)** Percentage of IL-17^+^ blood cells detected in CD4^+^CD3^+^ gated cells analyzed by flow-cytometry as described in Materials and Methods. Data are presented as percentages of cells among CD4^+^ T cells or by pg IL-6 per mL of serum or tumor supernatant. Data from 15 SD OSCC patients (■) were compared to those from 22 NSND OSCC patients (□). **(B)** Detection of IL-6 in serums from 15 SD (■) and 11 NSND (□) OSCC patients were compared to 5 NSND (○) and 13 SD (●) healthy donors. **(C)** Detection of IL-6 in tumor supernatants from 7 SD (■) and 4 NSND (□) OSCC patients. For statistical analysis, a Mann-Whitney test was used. Bars represent the standard error of the mean (SEM). *p ≤ 0.05; **p ≤ 0.01; ns: not significant.

Analysis of regulatory T cells (Treg) within CD4^+^ cells in both the TME and blood (**Figure 3**, lower left and middle right panels) from OSCC patients revealed a significant increase as compared to HD control groups (p < 0.0001), without difference between SD and NSND patients. Similarly, CD45RO^+^ CCR6^+^CD4^+^ cells (24, 25) (**Figure 3**, lower middle left and lower right panels) exhibited a significant increase in the TME (p = 0.0003) and blood (p = 0.0001) of SD patients, as compared to NSND patients and HD. Higher percentages of IL-17^+^ cells were also detected in the blood CD4^+^CD3^+^ T-cell compartment from SD patients as compared to NSND patients after *in vitro* PMA stimulation (**Figure 4A**). Of note, no difference (percentages and counts) was observed between SD and NSND healthy donors, both in gingival tissue and blood (**Figures 2B** and **3**). Thus, tobacco consumption associated or not with alcohol intake had no impact in healthy donors. Since CD45RO^+^CCR6^+^CD4^+^ cells contain cells that polarize towards an IL-17 phenotype upon *in vitro* stimulation and since Th17 cells promotes the secretion of IL-6 (25), we quantified IL-6 in sera from OSCC patients and HD and in tumor secretome. Significant higher levels were detected in SD patients as compared to NSND patients and to HD groups (**Figure 4B**), with the highest concentration observed in the tumor supernatants (**Figure 4C**).

**Figure 5:**
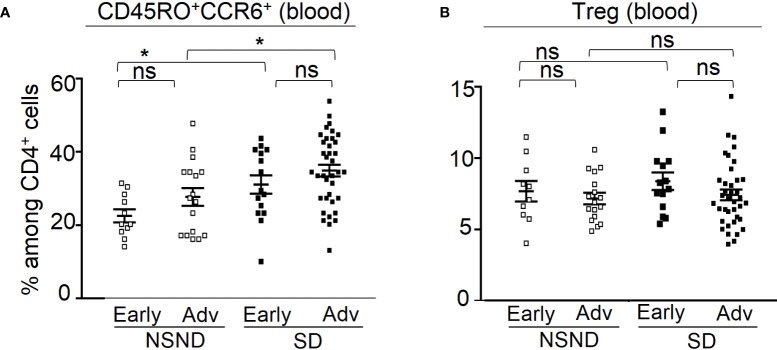
Analysis of circulating CD45RO^+^CCR6^+^ cells (left panel) and Treg (right panel) in patients at early and advanced OSCC stages. **(A)** The presence of circulating CD45RO^+^CCR6^+^ cells was analyzed in 28 NSND (□) (11 early and 17 advanced stages) and 52 SD (■) (15 early and 37 advanced stages) OSCC patients. **(B)** The presence of circulating Treg was analyzed in 27 NSND (□) (10 early and 17 advanced stages) and in 52 SD (■) (14 early and 38 advanced stages) OSCC patients were analyzed for Treg. Early stage: stages I and II; advanced stage (Adv): stages III and IV. For statistical analysis, a Mann-Whitney test was used. Bars represent the standard error of the mean (SEM). *p ≤ 0.05; ns: not significant.

Of note, a marked decrease in circulating CD4^+^ T cells in NSND patients was observed. Remarkably, whatever the tumor stage (early [I and II] and advanced [III and IV], the percentages of CD45RO^+^CCR6^+^ cells were increased in SD patients as compared to NSND patients (**Figure 5A**), whereas the percentages of Treg were similar (**Figure 5B**).

### 3.3 Tumor-infiltrating CD45RO^+^CCR6^+^CD4^+^ cells and Treg cells and blood CD45RO^+^CCR6^+^CD4^+^ cells and granulocytes are associated with the survival of SD patients but not of NSND patients

**Figure 6:**
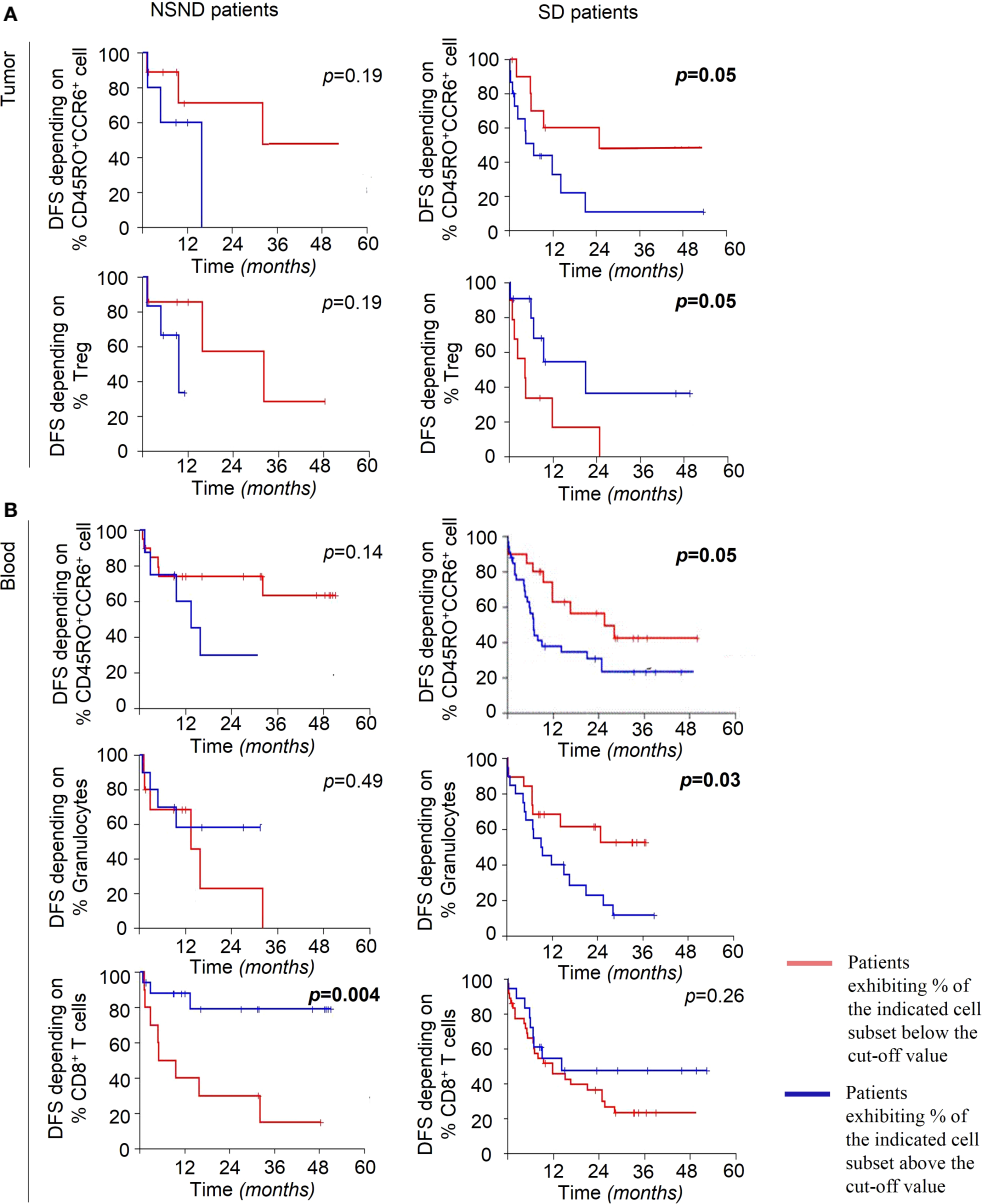
Disease-Free Survival (DFS) of OSCC patients. **(A)** DFS of NSND patients (left panels) and SD patients (right panels), dichotomized to the medians of percentages of CD45RO^+^CCR6^+^CD4^+^ T cells (19.2%) (upper panels) and Treg (18.6%) (lower panels) in TME. **(B)** DFS of NSND patients (left panels) and SD patients (right panels), dichotomized to the medians of CD45RO^+^CCR6^+^CD4^+^ cells (30%) (upper panels), of granulocytes (66%) (middle panels) and of CD8^+^ T cells (4.7%) (lower panels) in blood. The cut-off for each cell subset is the median value obtained for the whole cohort of patients (87 OSCC). Kaplan-Meier Disease-Free Survival curves are presented. The blue and red lines represent patients exhibiting percentages of the indicated cell population above and below the cut-off value, respectively. The Log-Rank test was used to calculate the differences observed between the groups of patients. p ≤ 0.05 was considered as significant.

Then, we performed an univariate COX regression analysis on both groups of patients to assess whether the differences observed in the blood and TME between SD and NSND patients are associated with different DFS (**Table 2** and **Figure 6A**). The presence of CD4^+^ or CD8^+^ T cells in the TME had no impact on the DFS of the whole cohort and for both groups of patients (**Table 2**). By contrast, the presence of more than 19.2% of CD45RO^+^CCR6^+^CD4^+^ cells was significantly associated with a poor prognosis in the TME of SD patients, but not in NSND patients (**Table 2** and **Figure 6A**, upper panels). The presence of more than 18.6% of Treg in the TME was a favorable prognostic factor for SD patients but not for NSND patients (**Table 2** and **Figure 6A**, lower panels).

**Table 2:** Univariate COX regression analysis of immune cells in OSCC patients.

			NSND patients		SD patients	
	Cell subset	Cut-off (%)^a^	HR (95% CI)^b^	p- value^c^	HR (95% CI)	p-value
**Blood**	Granulocytes	66	0.63 (0.16-2.36)	0.49	2.47 (1.05-5.80)	0.03
	CD8^+^ T cells	4.7	0.18 (0.048-0.68)	0.004	0.73 (0.36-1.45)	0.27
	CD4^+^ T cells	12	1.02 (0.27-3.84)	0.98	0.52 (0.26-1.07)	0.07
	Treg	7	0.58 (0.17-1.98)	0.37	0.86 (0.44-1.70)	0.67
	CD45RO^+^CCR6^+^ (CD4^+^ T cells)	30	2.38 (0.72-7.89)	0.14	2.09 (1.00-4.39)	0.05
**Tumor**	Granulocytes	29.8	2.76 (0.44-17.33)	0.26	0.605 (0.15-2.34)	0.46
	CD8^+^ T cells	9.4	0.41 (0.08-2.14)	0.28	0.80 (0.30-2.10)	0.64
	CD4^+^ T cells	13.8	2.05 (0.36-11.45)	0.40	0.53 (0.2-1.42)	0.20
	Treg	18.6	4.1 (0.42-40)	0.19	0.34 (0.11-1.07)	0.05
	CD45RO^+^CCR6^+^ (CD4^+^ T cells)	19.2	3.12(0.51-18.90)	0.19	2.79 (0.94-8.28)	0.05

^a^The cut-off for each cell subset is the median value obtained for the whole cohort of patients. ^b^HR, Hazard Ratio. 95% CI, 95% Confidence Interval. ^c^ The likelihood ratio test was used to calculate the differences observed between the groups of patients. p ≤0.05 was considered as significant. For a p-value ≤0.05, HR <1 or HR >1 are of favorable or poor prognosis, respectively.

In the blood from SD patients but not from NSND patients, the presence of more than 30% of CD45RO^+^CCR6^+^CD4^+^ cells or more than 66% of granulocytes were also significantly associated with a poor prognosis (**Table 2** and **Figure 6B**, upper and middle panels). In NSND patients, none of these markers except one (**Figure 6B**, left lower panel) had any incidence on the DFS (**Figure 6A** and **Figure 6B**, left panels). A percentage of CD8^+^ T cells greater than 4.7% appeared to be a better DFS for NSND patients but not for SD patients (**Table 2** and **Figure 6B**, lower panels).

Multivariate COX regression analyses confirmed that high percentages of blood granulocytes and CD45RO^+^CCR6^+^CD4^+^ cells are significantly associated with a poor prognosis in SD patients (**Table 3**).

**Table 3:** Multivariate COX regression analysis of blood granulocytes and CD45RO^+^CCR6^+^CD4^+^ T cell subset in SD OSCC patients.

	Cell subset	Cut-off (%)^a^	HR (95% CI)^b^	p-value^c^
**Blood**	Granulocytes	66	3.65 (1.43-9.28)	0.006
	CD45RO^+^CCR6^+^ (CD4^+^ T cells)	30	3.04 (1.21-7.67)	0.003

^a^The cut-off for each cell subset is the median value obtained for the whole cohort of patients. ^b^HR, Hazard Ratio. 95% CI, 95% confidence interval. ^c^p-value ≤0.05 was considered as significant. The likelihood ratio test was used to calculate the differences observed between the groups of patients. For a p-value ≤0.05, HR <1 or HR >1 are of favorable or poor prognosis, respectively.

## 4 Discussion

In the present report, we have set up a prospective cohort of OSCC patients to study whether their blood and TME immune cell contents are different. We show that SD patients exhibit a significant increase of CD45RO^+^CCR6^+^CD4^+^ cells and of IL-17^+^ cells, both in TME and blood, as compared to NSND patients and healthy donors, independently from the tumor stage, alongside with a significant higher level of IL-6, in both blood and tumor culture supernatants. The tobacco did not provoke *per se* this increase as it was not observed in SD healthy donors. Whether the comparison of SD patients with NSND patients is biased due to the presence of a high proportion of NSND patients (42.86%) belonging to the >70-year-old decade and beyond (**Table 1**) as compared to the SD patients (11.86%) is an open question. However, it should be stressed that, in our study, the age difference between NSND and SD patients is mostly related to a slight shift in the distribution of the highest number of patients in each group, from the > 70-year-old decade and beyond for NSND patients to the > 60-69-year-old decade for SD patients (**Figure 1**). Thus, the two groups of patients are closely age-related, making unlikely a significant impact on immune cell composition due to the difference.

In some experiments, we present the absolute number of immune cells per mg of tissue to give a whole view of the magnitude of tumor-infiltrating leukocytes in the different samples. This made it possible to examine whether differences in the magnitude of infiltration by immune cells between patients and healthy donors (**Figure 2A**) and between NSND and SD patients (**Figure 2B**) occur. Thus, we could show that immune cells infiltrating OSCC tumors are present in different numbers and proportions depending on the groups of patients (NSND vs SD). We have also evaluated the proportion of granulocytes among CD45^+^ cells in HD and OSCC patients. No significant differences were observed between the two groups of patients. This was expected since the OSCC patients exhibited an increase in the number of leukocytes, including granulocytes, leading to a lack of change in the Granulocytes/CD45^+^ ratio. Moreover, we analyzed cells isolated from tumor edge because this area is the region where immune cells are mostly present and exhibit strong cellular interactions in solid tumors including OSCC (15). It is well established that the centers of solid tumors are largely hypoxic and necrotic and contain fewer immune cells, making flow cytometry analysis of these cells difficult.

Two patients had an elevated percentage of IL-17^+^CD4^+^CD3^+^ cells. Both were males and SD, without any other disease or treatment. Their tumors were localized in gingiva and diagnosed at a late stage (Stade 4). These two patients were not excluded from statistical analyses. First, they were studied in the same experiments using the same protocol and reagents as other patients. Thus, there is no obvious experimental bias. Second, regarding to their clinical status, no parameter (gender, stage of the disease, tumor localization) distinguished them from other patients. Interestingly, these two patients had Stage 4 OSCC. This stage has been associated with a marked change in the oral microbiota environment, with a significant increase in the presence of various bacteria (26). Notably, the presence of F. nucleatum has been correlated with an increase in the IL-17A production and in the frequency of intestinal Th17 cells in colorectal cancer (27). Also, in OSCC, both F. nucleatum and P. gingivalis provoke an increase of the local levels of IL-6 and CCL20 (26). Thus, assessing the bacterial load in the OSCC-induced lesions depending on the NSND and SD status is certainly an important investigation to be done.

Of note, a study based on genomic data has suggested that SD and NSND patients exhibit different transcriptomic profiles of TME (13) in line with our present results. The increase of CD45RO^+^CCR6^+^CD4^+^ cells, IL-17^+^ cells and granulocytes in SD patients was significantly associated with a poor prognosis, although no difference in terms of the overall DFS between SD and NSND patients was observed, as already reported (28–30). Whether the increased presence of Th17^+^ cells is associated with a poor prognosis is still a matter of controversy. In an heterogenous cohort of HNSCC patients, it has been shown that blood IL-17^+^ cells are negatively correlated with the overall survival (31). Also, in OSCC patients, the detection of IL-17 in tumor budding was found to be associated with a poor prognosis (32). By contrast, it has been reported that Th17 cells which are present in large numbers both in the peripheral blood and TME of HNSCC patients, inhibit the proliferation of HNSCC cells (33). Also, in OSCC, it has been suggested that Th17 cells may have effector immune functions in oral cancer immunity (21).

The mechanism by which CD45RO^+^CCR6^+^CD4^+^ cells and granulocytes are recruited in TME is likely related to the presence of elevated amounts of IL-6 in tumor secretome from SD OSCC patients. Among pro-inflammatory cytokines, IL-17 triggers the accumulation in blood and tissues of neutrophils, major source of IL-6, a potent pro-inflammatory cytokine known to polarize CD4^+^ T cells toward a Th17 cell phenotype (34–36). Th17 cells in turn can recruit granulocytes, mostly neutrophils and eosinophils (37) that are strongly involved in tumor immune surveillance. Their pro-tumor role in TME has been highlighted although they can also exert an anti-tumor activity by direct killing of tumor cells and through their participation in the activation of anti-tumor cellular networks (38). Of note, a significant difference in plasma levels of IL-6 was found between early and advanced OSCC stages (19). Also, it has been reported that IL-6 increases the migration properties of OSCC cells (39). In our work, no elevation of IL-6 level was observed in NSND patients, together with a lack of significant increase of CD45RO^+^CCR6^+^CD4^+^ cells and of IL-17^+^ cells in their TME and blood.

The proportion of B and T lymphocytes in the tumors were not different from those of tissue-infiltrating lymphocytes in healthy donors. However, an increase of the frequency of blood CD8^+^ T lymphocytes was associated with a better prognosis for NSND patients but not SD patients. A high frequency of CD4^+^ T cells either in TME or blood had no impact on the prognosis in both groups of patients, in agreement with some reports (40, 41) but not all (42). Actually, a meta-analysis about the prognostic significance of CD4^+^ and CD8^+^ tumor-infiltrating lymphocytes in oral cavity cancers showed that the prognostic relevance depends on their intra-tumoral location (43).

A significant increase of Treg was also observed in the TME and blood from SD OSCC patients, as compared to SD healthy donors and NSND patients. The presence of more than 18% of Treg in TME was a favorable prognostic factor only for SD OSCC patients by contrast to NSND patients. Although high levels of Treg in TME or blood are usually associated in most cancer patients with a poor prognosis (44), their inhibitory effect on suppressive myeloid cells and pro-inflammatory cells that participate to tumor development such as Th17 cells could also hinder tumor growth and metastatic dissemination. This has been exemplified in pancreatic tumor where Treg depletion in mice has led to an increase of immunosuppressive myeloid cells and fibroblast reprogramming and accelerated carcinogenesis (45). Furthermore, an increase of Treg has been often associated with a more favorable prognosis in HNSCC, including OSCC (46) as observed in the present study.

A follow-up of the immune cell populations relative to the continuation of exposure to risk factors (smoking/drinking) during treatment of OSCC SD patients was not performed in the study. It should be of interest to do it in further studies where a larger group of SD patients will be studied. Notably, it has been demonstrated that tobacco consumption during external beam radiotherapy of prostate cancer negatively impacts prognosis (47) whereas cessation after diagnosis of lung cancers improves prognostic outcomes (48).

Overall, our work shows that blood and TME immune cells in NSND and SD OSCC patients present a different phenotypic profile. Specific biomarkers for SD patients (i.e., CD45RO^+^CCR6^+^CD4^+^ cells, granulocytes, monocytes-macrophages, and Treg) and for NSND patients (CD8^+^ T cells) have been identified as strong candidates for being prognostic biomarkers. Future studies based on larger cohorts of NSND and SD OSCC patients should strengthen the data presented herein. Such studies should discriminate former smokers from strictly non-smokers and blood analysis should be performed during the follow-up of the patients. The biomarkers evidenced in the present study could be then used in routine clinical practice to evaluate possible tumor recurrence and identify patient subgroups eligible to immunotherapies.

## Data availability statement

The original contributions presented in the study are included in the article/**Supplementary Material**. Further inquiries can be directed to the corresponding author.

## Ethics statement

The studies involving human participants were reviewed and approved by Ethics Committee of Inserm (n°19-564).. The patients/participants provided their written informed consent to participate in this study.

## Author contributions

JR: acquisition of data, analysis and interpretation of data, drafted the manuscript and critically revised the manuscript for important intellectual content. IK and CIB: acquisition and analysis of data. LB: analysis of data. MG-D: Analysis of data and critically revised manuscript. RM: acquisition of data. AL: acquisition of data. VM: analysis of data. PS: acquisition of data. IB: acquisition of data. J-LT: draft the manuscript and critically revised the manuscript for important intellectual content. M-CD-N: critically revised the manuscript for important intellectual content. CB, F-ML, and GL: conception and design, analysis and interpretation of data, drafted the manuscript and critically revised the manuscript for important intellectual content. All authors contributed to the article and approved the submitted version.

## Supplementary material

Supplementary Figure 1 Gating strategy used in the study of tumor samples and whole blood. After dead cells (for tumor samples) and doublets exclusion, seven subpopulations gated on CD45^+^ cells were identified: CD15^+^CD11b^+^ (granulocytes), CD14^+^ (monocytes-macrophages (Mo-Mϕ), CD19^+^CD3^-^ (B cells), CD3^+^CD4^+^ (CD4^+^ T cells), CD3^+^CD8^+^ (CD8^+^ T cells), CD3^+^CD4^+^CD25^+^FoxP3^high^CD127^low^ (Treg), CD45^+^CD4^+^CD45RO^+^CCR6^+^ (CD4^+^ T cell subset containing cells able to polarize towards a Th17 phenotype upon *in vitro* stimulation).
