# Supplementary material for: Defining biomarkers in oral cancer according to smoking and drinking status
Source: Front Oncol. 2023 Jan 11;12:1068979. doi: 10.3389/fonc.2022.1068979 (PMC9875375; doi:10.3389/fonc.2022.1068979)
Supplement: Supplementary file 1 [file Table_1.docx]

| Characteristics | | HD^a^  n=71 | NSND^b^ HD  n=28 | SD^c^ HD  n=43 | p*-*value |
| --- | --- | --- | --- | --- | --- |
| Gender | Male | 39 (54.93%) | 14 (50%) | 25 (58.14%) | 0.5005 |
|  | Female | 32 (45.07%) | 14 (50%) | 18 (41.86%) |  |
| Age | Min/Max^d^ | 15 /87 | 15/87 | 20/87 | 0.4099 |
|  | Med^e^  [Q1-Q3^f^] | 43  [31.5-58] | 38.5  [27.5-58] | 44  [33-58] |  |
|  | <50 years | 42 (59.15%) | 17 (60.71%) | 25 (58.14%) | 1.0000 |
|  | 50-70 years | 21 (29.58%) | 8 (28.57%) | 13 (30.23%) |  |
|  | >70 years | 8 (11.27%) | 3 (10.71%) | 5 (11.63%) |  |

**Supplementary Table 1.**  Age and gender distribution of Non-Smoker/Non-Drinker (NSND) and Smoker/Drinker (SD) healthy donors (HD).

a: HD, healthy donors; b: NSND, Non-Smoker/Non-Drinker; c: SD, Smoker/Drinker; d: Min/Max, minimum-maximum; e: Med, median; f: Q1-Q3, Quartile 1 (lower quartile)-Quartile 3 (upper quartile). Qualitative variable analyses were performed by a chi-square test.
