# Supplementary material for: Defining biomarkers in oral cancer according to smoking and drinking status
Source: Front Oncol. 2023 Jan 11;12:1068979. doi: 10.3389/fonc.2022.1068979 (PMC9875375; doi:10.3389/fonc.2022.1068979)
Supplement: Supplementary file 2 [file Table_2.docx]

**Supplementary Table 2.** List of monoclonal antibodies used.

| **mAbs & fluorochromes** | **Host reactivity** | **Clone** | **Manufacturer** |
| --- | --- | --- | --- |
|  |  |  |  |
| CD3-BV 650 | Mouse anti-human | UCHT1 | BD Pharmigen™ |
| CD4-VioBlue® | Mouse anti-human | VIT4 | Miltenyi Biotec |
| CD8-VioGreen™ | Mouse anti-human | BW135/80 | Miltenyi Biotec |
| CD11b-FITC | Mouse anti-human | M1/70.15.11.5 | Miltenyi Biotec |
| CD14-PE-Vio770™ | Mouse anti-human | TÜK4 | Miltenyi Biotec |
| CD15-APC | Mouse anti-human | VIMC6 | Miltenyi Biotec |
| CD19-FITC | Mouse anti-human | LT19 | Miltenyi Biotec |
| CD19-PerCP-eFluor®710 | Mouse anti-human | J3-129 | eBioscience |
| CD25-PE | Mouse anti-human | 4E3 | Miltenyi Biotec |
| CD45-AlexaFluor®700 | Mouse anti-human | H130 | BD Pharmigen™ |
| CD45RA-APC | Mouse anti-human | T6D11 | Miltenyi Biotec |
| CD45RO- eFluor®650NC | Mouse anti-human | UCHL1 | eBioscience |
| CD127- PE-Vio770™ | Mouse anti-human | MB15-18C9 | Miltenyi Biotec |
| CD196-APC (CCR6) | Mouse anti-human | 11A9 | BD Pharmigen™ |
| FoxP3-APC | Mouse anti-human | 236A/E7 | eBioscience |
| IL17a-eFluor 660 | Mouse anti-human | 64CAP17 | eBioscience |
| Live/Dead-APCH7 |  |  | Invitrogen™ |

*PE : Phycoerythrin; FITC : Fluorescein isothiocyanate ; APC : Allophycocyanin ; PerCP : Peridinin Chlorophyll Protein ; BV : Brillant Violet.
