# Supplementary figures and images for: Defining biomarkers in oral cancer according to smoking and drinking status
Source: Front Oncol. 2023 Jan 11;12:1068979. doi: 10.3389/fonc.2022.1068979 (PMC9875375; doi:10.3389/fonc.2022.1068979)

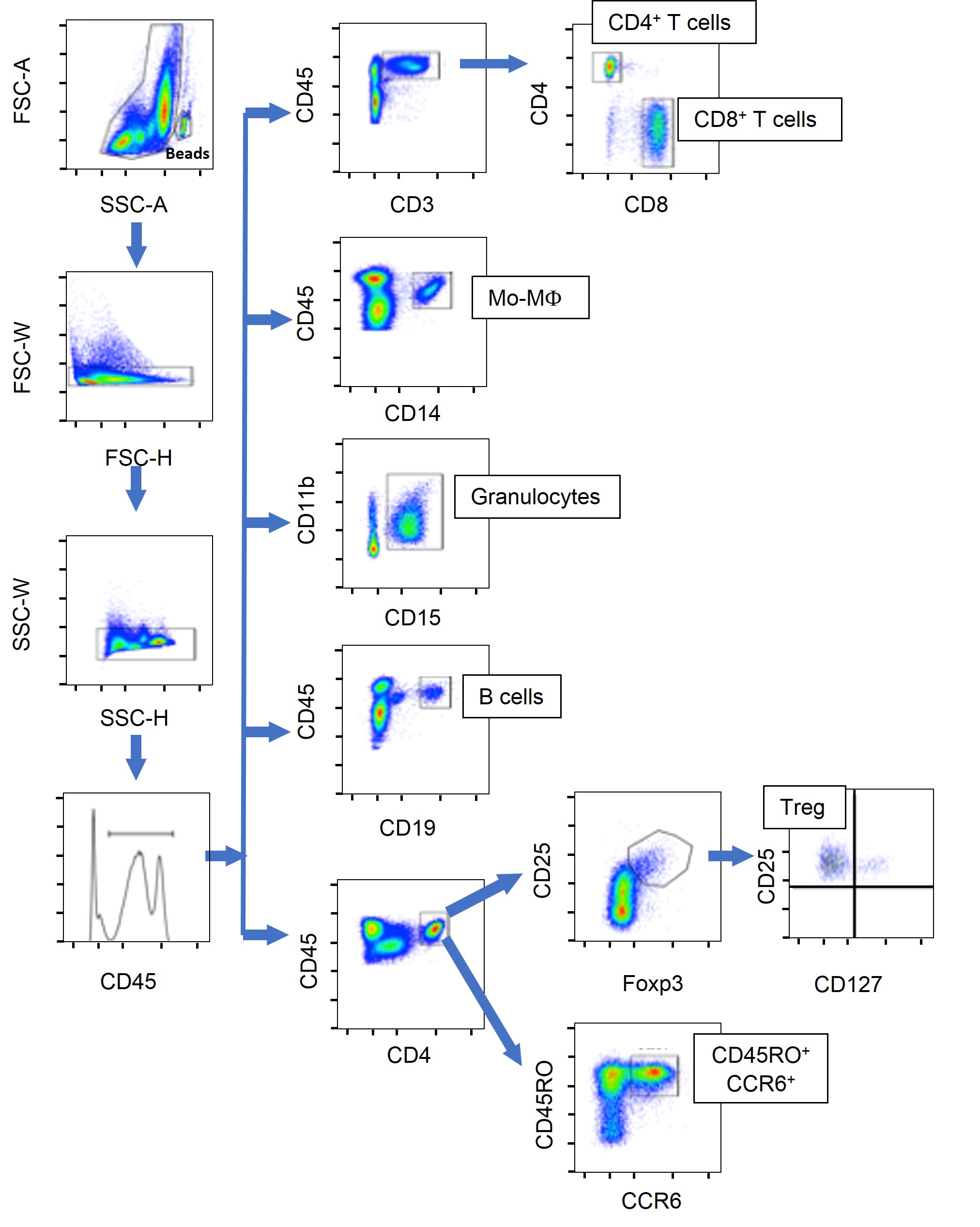

Supplement: Supplementary Figure 1 — Gating strategy used in the study of tumor samples and whole blood. After dead cells (for tumor samples) and doublets exclusion, seven subpopulations gated on CD45+ cells were identified: CD15+CD11b+ (granulocytes), CD14+ (monocytes-macrophages (Mo-Mϕ), CD19+CD3- (B cells), CD3+CD4+ (CD4+ T cells), CD3+CD8+ (CD8+ T cells), CD3+CD4+CD25+FoxP3highCD127low (Treg), CD45+CD4+CD45RO+CCR6+ (CD4+ T cell subset containing cells able to polarize towards a Th17 phenotype upon in vitro stimulation). [file Image_1.jpeg]
